# Endochondral Ossification for Spinal Fusion: A Novel Perspective from Biological Mechanisms to Clinical Applications

**DOI:** 10.3390/jpm14090957

**Published:** 2024-09-09

**Authors:** Rile Ge, Chenjun Liu, Yuhong Zhao, Kaifeng Wang, Xiluan Wang

**Affiliations:** 1 Department of Orthopedics, Beijing Friendship Hospital, Capital Medical University, No. 95, Yong An Rd, Beijing 100050, China; gerile@pkuph.edu.cn; 2 Department of Spinal Surgery, Peking University People’s Hospital, 11th Xizhimen South Ave., Beijing 100044, China; liuchenjun@bjmu.edu.cn; 3 Beijing Key Laboratory of Lignocellulosic Chemistry, Beijing Forestry University, Beijing 100083, China; zyh925994@bjfu.edu.cn

**Keywords:** degenerative scoliosis, endochondral ossification, spinal fusion, growth plate, bone repair, 3D printing personalized interbody fusion cages, polyetheretherketone (PEEK)

## Abstract

Degenerative scoliosis (DS), encompassing conditions like spondylolisthesis and spinal stenosis, is a common type of spinal deformity. Lumbar interbody fusion (LIF) stands as a conventional surgical intervention for this ailment, aiming at decompression, restoration of intervertebral height, and stabilization of motion segments. Despite its widespread use, the precise mechanism underlying spinal fusion remains elusive. In this review, our focus lies on endochondral ossification for spinal fusion, a process involving vertebral development and bone healing. Endochondral ossification is the key step for the successful vertebral fusion. Endochondral ossification can persist in hypoxic conditions and promote the parallel development of angiogenesis and osteogenesis, which corresponds to the fusion process of new bone formation in the hypoxic region between the vertebrae. The ideal material for interbody fusion cages should have the following characteristics: (1) Good biocompatibility; (2) Stable chemical properties; (3) Biomechanical properties similar to bone tissue; (4) Promotion of bone fusion; (5) Favorable for imaging observation; (6) Biodegradability. Utilizing cartilage-derived bone-like constructs holds promise in promoting bony fusion post-operation, thus warranting exploration in the context of spinal fusion procedures.

## 1. Introduction

Degenerative scoliosis (DS) represents a prevalent form of spinal deformity, particularly manifesting with age-related trends. This condition predominantly affects adults, typically emerging amidst skeletal maturity, and arises from pronounced degeneration of intervertebral discs and bilateral facet joints, primarily in the thoracolumbar or lumbar regions. Consequently, patients often experience lower back pain, exacerbated by concurrent spinal canal stenosis, which can induce radiating pain in the lower extremities, impairing mobility and substantially diminishing overall quality of life [1,2].

Among the array of surgical interventions for DS, posterior lumbar interbody fusion (PLIF) stands out as a promising approach, which aims to effectively decompress spinal canals, restore intervertebral heights, and stabilize motion segments that cause discomfort. Notably, the utilization of interbody fusion cages in posterior lumbar fusion surgery represents a well-established method for managing degenerative lumbar conditions. These cages afford superior segmental stability and establish an optimal milieu for promoting bone implantation, thereby preserving intervertebral height and stability [3,4].

However, achieving a satisfactory spinal fusion rate necessitates additional supplementation of the internal and/or surrounding environment of the interbody fusion cage with various graft materials that possess osteogenic, osteoconductive, and osteoinductive properties. These materials include autogenous bone, cancellous bone, bone marrow, allograft bone, and bone morphogenetic protein (BMP). Despite advancements, the precise mechanism underlying spinal fusion remains ambiguous. Recent evidence suggests endochondral ossification plays a pivotal process in the fusion cascade [5,6].

In this review, we discuss the intricate realm of endochondral ossification for spinal fusion, spanning from elucidating its biological mechanisms to exploring its clinical applications in mitigating degenerative scoliosis and related conditions.

## 2. The Mechanisms for Vertebrae Growth

Following birth, the formation of the spine includes nine secondary ossification centers, such as the superior and inferior articular processes, transverse processes, spinous processes, as well as the superior and inferior growth plates (GPs). These secondary ossification centers play an important role in facilitating the longitudinal growth of the spine via the process of endochondral ossification [7,8]. The typical GP can be anatomically divided into distinct zones, including the resting zone, proliferative zone, hypertrophic zone, and calcification zone [9]. Longitudinal spine growth predominantly occurs through the orchestrated activities within these zones. Specifically, this process entails the proliferation of chondrocytes within the proliferative zone, subsequent enlargement of chondrocytes within the hypertrophic zone, and the concomitant formation of the cartilage matrix [10].

The GP, also referred to as the epiphysis, constitutes the cartilaginous segment situated at the extremities of long bones, serving as a pivotal locus for initiating and regulating longitudinal skeletal growth. Functionally dynamic and intricately structured, the GP primarily resides at the metaphysis and vertebral body of long bones [11]. Derived from mesenchymal cells (MSCs) originating from the mesoderm, the cartilaginous tissue within the GP undergoes organization into distinct zones, including the resting zone, proliferative zone, and hypertrophic zone. Microscopically, it is characterized by chondrocytes embedded within a collagen matrix. During the process of longitudinal bone growth, chondrocytes within the GP go through a series of sequential transformations, including differentiation, maturation, and eventual apoptosis, before being replaced by osteoblasts, osteoclasts, and the formation of trabecular bone [12,13,14].

In general, bone formation in the GP begins with pre-chondrogenic MSCs, which differentiate into proliferative chondrocytes and metabolically active hypertrophic chondrocytes within the GP [15]. During chondrocyte proliferation and hypertrophic differentiation, their endoplasmic reticulum synthesizes extracellular matrix (ECM) components and proteins, inducing osteoblast differentiation and subsequent bone formation [16]. Pre-chondrogenic MSCs aggregate into cell clusters under the influence of adhesion molecules. Some cells directly differentiate into osteoblasts post-clustering, dispersing within type I collagen, termed intramembranous bone formation [17]. Conversely, in most cases, pre-chondrogenic MSCs differentiate into chondrocytes, which, upon hypertrophic differentiation, secrete ECM, facilitating bone formation via endochondral ossification [18]. Chondrocyte differentiation and proliferation follow five classical stages: resting, proliferative, prehypertrophic, hypertrophic, and terminal phases [19].

During the resting phase, chondrocytes display relatively inactive mitotic activity but express the *COL2A1* gene, which encodes type II collagen. While in the proliferative phase, chondrocytes divide quickly and express *COL2A1* and the *ACAN* gene, which codes for the proteoglycan aggrecan, at high levels. Following rapid proliferation, chondrocytes progress to the hypertrophic phase. Here, they begin expressing the *COL10A1* gene, encoding type X collagen, and the *IHH* gene, encoding the Indian Hedgehog protein. Subsequently, as chondrocytes continue through the hypertrophic phase, their volume increases, with ongoing expression of type X collagen while the expression of type II collagen ceases. Upon completion of the hypertrophic phase, chondrocytes cease synthesizing all collagen products and enter programmed cell death in the terminal phase [20,21].

After passing through the proliferative and hypertrophic phases, chondrocytes in the GP produce a substantial number of cartilage ECM components, resulting in the formation of new cartilage tissue. Following this, via processes such as cartilage ECM calcification, the tissue is transformed into bone tissue, resulting in longitudinal bone development and changes in children’s height. During childhood, the GP diameter steadily decreases due to estrogen. After all of the chondrocytes in the GP die, the cartilage tissue is replaced by mature bone tissue, leaving linear remnants known as epiphyseal lines. The closure of the epiphysis signals the conclusion of the longitudinal bone development process [22,23].

The cartilaginous tissue within the GP exhibits elevated metabolic activity and is subject to regulation by various hormones and signaling pathway molecules [24]. It is reported that the proliferation and differentiation of chondrocytes within the GP are influenced by factors such as nutritional elements, inflammatory mediators, extracellular fluid composition, ECM, and endocrine agents [25]. In addition to the classical GH-IGF-1 axis, numerous basic and clinical investigations have revealed that the regulation of cartilage formation within the GP necessitates the involvement of multiple signaling pathways [26]. These pathways include the transforming growth factor-β (TGF-β)/BMP signaling pathway, Wnt/β-catenin signaling pathway, fibroblast growth factor (FGF) signaling pathway, Hedgehog (HH) signaling pathway, and Notch signaling pathway [27,28,29,30]. The growth and development of GP cartilage constitute a meticulously orchestrated biological process governed by a combination of ECM composition, intracellular signals, paracrine signaling molecules, endocrine signals, and other factors [9,31]. Based on the biological functions attributed to different signaling pathways, they can be categorized into four principal regulatory mechanisms: hormonal signaling [32], paracrine signaling [33], ECM maintenance signaling [34], and fundamental cellular processes [35].

## 3. The Mechanisms for Bone Repair

**Figure 1:**
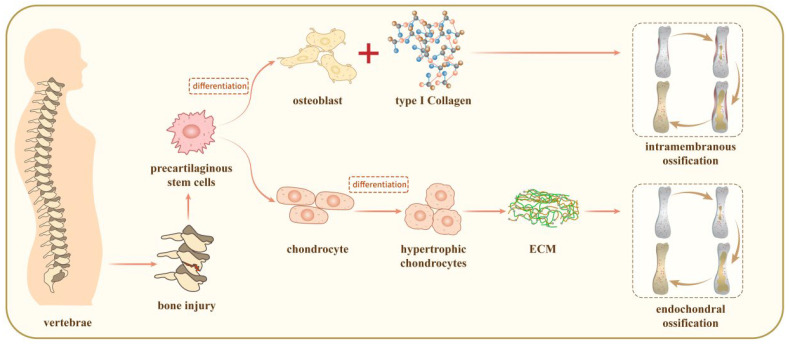
Intramembranous ossification and endochondral ossification.

Bone repair modalities include intramembranous ossification and endochondral ossification (Figure 1). In intramembranous ossification, MSCs directly differentiate into osteoblasts under the defective proximal periosteum to produce bone tissue. Endochondral ossification is far more complicated than intramembranous ossification. MSCs first differentiate into chondrocytes and secrete cartilage matrix at the bone injury, forming a cartilage prototype to connect the fracture ends, followed by chondrocyte hypertrophy and calcification, and thereafter, osteoblasts grow into the area to decompose and resorb the calcified cartilage matrix, while vascularity and osteoclasts enter into the area, and the cartilage is gradually replaced with bone [36,37,38,39].

**Figure 2:**
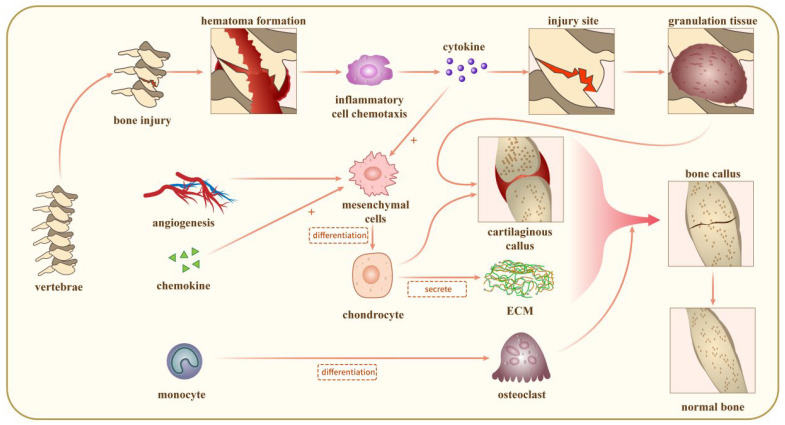
The mechanisms for bone repair in the spine.

As far as the human body is concerned, certain bones, such as the frontal bone, parietal bone, and clavicle, are repaired by intramembranous ossification. In contrast, the repair of most irregular bones, such as long bones, short bones, and jaw bones, is based on endochondral ossification [40]. It can be seen that endochondral ossification is a very important repair mode in organic bone injury, especially for large bone defects (Figure 2). The formation of cartilage tissue plays a role in mechanically repairing the fracture breaks and providing scaffolding for the deposition of bone matrix [41]. Moreover, chondrocytes are more tolerant to hypoxic environments than MSCs and osteoblasts [42]. Therefore, more and more studies are now focusing on bone regeneration and repair of endochondral osteoblasts, which has a wide range of applications in the field of bone regeneration [43].

### 3.1. Acute Inflammatory Response

The hematoma formation and the consequent acute inflammatory response that occurs after a bone injury initiates the bone repair process [44,45]. Accompanying the bone injury, blood vessels within the surrounding tissue and bone marrow rupture to form a hematoma, leading to exposure of platelets to the extravascular environment, and the blood begins to coagulate to form a fibrin network. The fibrin network formed by the clotting of the hematoma is a temporary site for inflammatory cells to reside [46]. Within 24 h, neutrophils migrate first to the injury and recruit a second wave of monocytes and macrophages to migrate to the fracture site [47]. Macrophages migrating to the site of bone injury play an important role in the whole bone repair process [48]. Firstly, macrophages help to remove cellular debris as well as possible pathogenic microorganisms from the injury site through phagocytosis. Secondly, macrophages are able to produce a variety of pro-inflammatory and chemotactic factors, such as tumor necrosis factor-α (TNF-α), interleukin-1β (IL-1β), IL-6, and CCL2, which amplify the inflammatory response, recruiting more inflammatory cells, fibroblasts, mesenchymal stromal cells, and bone marrow stem cells to migrate to the injury site. Finally, when pathogenic microorganisms and necrotic cell debris are removed, macrophages can produce growth factors such as transforming growth factor-β (TGF-β), bone morphogenetic proteins (BMPs), vascular endothelial growth factor (VEGF), and fibroblast growth factor-2 (FGF-2) to promote tissue regeneration at the injury site [49,50]. With the combined effects of these cells and factors, the initial hematoma and subsequent acute inflammatory response are cleared within a few days to 1 week, the injury site is replaced by granulation tissue rich in MSCs, and neovascularisation develops and grows into the disorganized extracellular collagen matrix [51].

### 3.2. Recruitment of MSCs

Bone regeneration first requires specific MSCs to migrate to the site of the bone injury, proliferate and differentiate into osteoblasts, and then undergo bone repair [52]. Where exactly these MSCs come from is not yet conclusive. In one study, bone marrow-derived MSCs were transfected with luciferase and injected into mice via the tail vein. It was found that the transfected cells migrated through the circulatory system to the site of the tibial fracture and participated in bone repair, so it is believed that circulating MSCs may be very important for bone healing. Some studies have also specifically labeled periosteal-derived MSCs by transgene labeling and observed that most of the cells in the bone scab at the tibial fracture site were derived from periosteal MSCs, confirming that periosteal MSCs are the main source of bone injury healing. The mechanism of migration of MSCs to the site of the bone injury has not yet been fully elucidated, and cytokines and chemokines may play an important role in this process [53]. Stromal cell-derived factor 1 (SDF-1) belongs to the CXC family and can specifically bind to cells expressing chemokine receptor-4 (CXCR-4) [54]. Hypoxia develops at the site of the bone fracture, and tissue damage brought on by hypoxia may boost the release of chemokines [55]. Together, these studies suggest that the SDF-1/CXCR4 axis may play a key role in cell migration and differentiation at the fracture site. Therefore, increasing the expression of CXCR4 ligands on the surface of MSCs is expected to enhance the migration and differentiation of MSCs and promote bone repair [56]. In addition to SDF-1, TGF-β, BMP2, and BMP7 were also involved in the homing of MSCs [55,56,57].

### 3.3. Cartilaginous Callus Generation

During the process of endochondral ossification, MSCs are recruited to the site of injury in the early stages of bone damage (day 1), proliferating and differentiating into chondrocytes around day 3, forming a cartilaginous callus. In animal models such as rats, mice, and rabbits, cartilaginous callus formation peaks at 7–10 days after fracture, primarily occurring in the fracture gap that is hypoxic, mechanically unstable, and distant from the fracture ends [58,59]. The environment in these areas is complex due to local vascular disruption and arteriolar reactive constriction caused by the fracture, resulting in hypoxia in the surrounding environment, especially near the fracture gap [60]. Under the combined action of slight movements, various microenvironments, and signaling molecules, MSCs differentiate into chondrocytes, proliferate, and form ECM, particularly at the central position of the fracture gap [61,62]. Several weeks later, the cartilage produced by chondrocytes extends throughout the entire fracture gap, serving as a bridge to connect the bone defect. The cartilage generated at this stage is not mineralized. It is surrounded by fibrous tissue, known as a cartilaginous callus, which gives initial mechanical support to the fracture and acts as a scaffold for endochondral bone development [63,64].

### 3.4. Revascularization

Blood supply is required for bone defect healing because blood vessels not only give oxygen and nutrition but also calcium and phosphate, which are necessary for cartilage matrix mineralization. Additionally, blood vessels create a favorable microenvironment for MSCs and hematopoietic stem cells. Inadequate blood supply is considered a major cause of delayed or non-union of bone defects. The regeneration of blood vessels in bone repair is regulated by various signaling pathways and growth factors, with VEGF being recognized as a primary regulator of angiogenesis [65]. Its secretion is regulated by multiple factors, with hypoxia being an important regulator of VEGF expression, particularly in tumors and bone tissue. VEGF promotes the establishment of blood flow to bring more MSCs to the fracture area and stimulates endothelial cells to produce cytokines that promote the differentiation of progenitor cells into osteoblasts, directly influencing the function of osteoblasts and directly or indirectly participating in bone regeneration [66,67].

### 3.5. Mineralisation and Resorption of Cartilaginous Calluses

During the process of differentiation from MSCs to chondrocytes, precise regulation by the transcription factor Sox9 is exerted [68]. Chondrocytes exhibit significant proliferative activity and specifically express chondrocyte markers such as type II collagen and aggrecan. In studies of rat fracture repair, at 10–14 days post-injury, proliferating chondrocytes gradually differentiate into hypertrophic chondrocytes (HCs) [69]. During this stage, the cell volume significantly increases, reaching 5 to 10 times that of proliferating phase cells. At this critical stage, mature HCs specifically express type X collagen and a series of characteristic markers of osteoblasts, such as alkaline phosphatase, osteocalcin, osteopontin, Osx, and Runx2, and possess the ability to produce matrix vesicles [70,71]. These matrix vesicles are released into the ECM and actively participate in the mineralization process. With the terminal differentiation of HCs, the process of mineralized cartilage matrix absorption is initiated. This differentiation process is mainly mediated by epidermal growth factor receptor (EGFR) and reactive oxygen species (ROS), characterized by a gradual decrease in type X collagen synthesis and the simultaneous production of a series of metalloproteinases (MMPs), among which MMP13 plays a major role, along with MMP9 and VEGF [72]. The degradation of the mineralized cartilage matrix provides space for vascular invasion. Consequently, a large number of blood vessels grow into the stable cartilage scaffold interstices, increasing blood supply to the site of fracture healing, carrying more MSCs and monocyte-macrophages [73,74]. Subsequently, MSCs differentiate into osteoblasts, which deposit woven bone on the remaining cartilage scaffold. In contrast, monocyte-macrophages differentiate into osteoclast-like cells, assisting in the absorption of the remaining mineralized cartilage matrix and immature new bone [75]. This process results in the gradual replacement of mineralized cartilage matrix by woven bone with trabecular structure, forming a hard callus, a process that typically lasts for weeks to months.

### 3.6. Bone Remodeling

With the formation of the hard callus, the bone defect is gradually filled with new bone, and the initial stability is improved. However, due to the irregular arrangement of bone trabeculae and the abundance of osteocytes, multiple bone remodeling processes are required to increase the number of bone units and to arrange the bone trabeculae according to tension and stress to meet the body’s movement and weight-bearing demands [76,77]. Bone remodeling is a process of continuous bone resorption and formation, and its specific mechanism is still under exploration. It is suggested that bone remodeling is accomplished through the perception of mechanical stimuli by multicellular units, which include osteoclasts, osteoblasts, and bone cells, and the cycle is divided into three stages: activation, resorption, and formation [78,79].

During the activation stage, surrounding bone cells and osteoblasts release cytokines, recruiting mononuclear macrophages from the bloodstream to the bone resorption site and differentiating them into osteoclasts [80]. Among them, osteoblast-secreted macrophage colony-stimulating factor (M-CSF) and RANKL can promote the differentiation of mononuclear macrophages into osteoclasts, while osteoprotegerin (OPG) secreted by them can inhibit the function of RANKL [81]. Bone cells can also regulate osteoclast differentiation by secreting RANKL and scleroprotein. In addition, other cytokines such as parathyroid hormone (PTH), parathyroid hormone-related peptide (PTHrP), TNF-α, and IL-1β can participate in the regulation of osteoclast differentiation by regulating the expression of RANKL in osteoblasts [82]. Through the continuous cycle of these three stages, the hard callus is eventually remodeled into bone with a normal structure that adapts to the body’s movement and weight-bearing, a process that lasts from months to years.

Despite extensive exploration into the mechanisms of endochondral ossification, understanding the role of neutrophils in acute inflammation, the source and migration mechanism of MSCs in bone injury repair, the mineralization mechanism and ultimate fate of hypertrophic chondrocytes, and the coupling mechanism of bone remodeling remains very limited and requires further investigation [59,83].

## 4. Endochondral Ossification and Bone Graft Substitutes for Lumbar Interbody Fusions

Endochondral ossification is the key step for successful vertebral fusion. In spinal fusion, previous studies have described new bone formation through a process of intramembranous ossification and endochondral ossification, which is similar to long bone fracture healing. Endochondral ossification supports bone formation and renewal. The process involves mesenchymal stem cells (MSCs). The aggregation of MSCs differentiates into primary chondrocytes, which in turn synthesize galactosamine sugars and cartilage-specific matrix proteins. The subsequent maturation stage witnessed the proliferation of chondrocytes, and their terminal differentiation was marked by hypertrophy, X-type collagen expression, and stromal calcification. It is noteworthy that endochondral ossification can persist in hypoxic conditions and promote the parallel development of angiogenesis and osteogenesis, which corresponds to the fusion process of new bone formation in the hypoxic region between the vertebrae [84]. By mimicking the local microenvironment, MSCs involved in endochondral osteogenesis may provide a viable pathway for new bone formation. Therefore, the development of an engineered endochondral osteogenic model that is fully adapted to the surgical needs of spinal fusion by focusing on the local improvement of endochondral osteogenic capacity is expected to promote bone regeneration and bone reconstruction in the vertebral space [85,86].

The interbody fusion cage combined with the pedicle screw-rod system is a common surgical method for treating degenerative diseases of the lumbar spine, such as lumbar spinal stenosis, spondylolisthesis, lumbar disc herniation combined with lumbar instability etc., including posterior lumbar interbody fusion (PLIF) and transforaminal lumbar interbody fusion (TLIF), has achieved good results [87].

**Figure 3:**
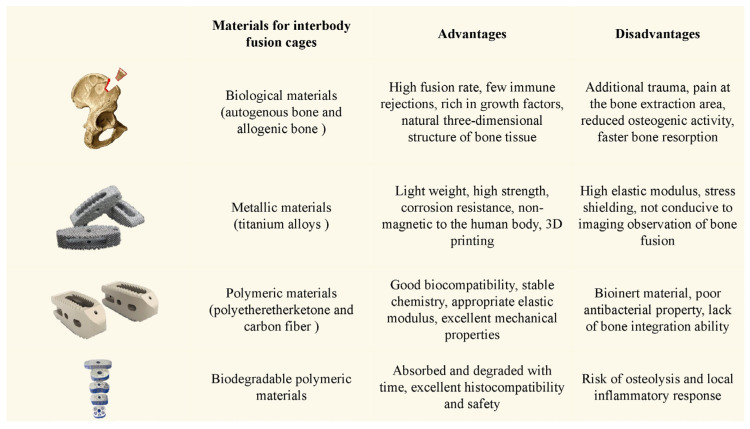
Four main types of materials for interbody fusion cages.

There are four main types of materials for interbody fusion cages (Figure 3): (1) Biological materials, mainly referring to autogenous bone and allogeneic bone. The lumbar intervertebral fusion rate of autogenous bone is extremely high because it has almost no infectious or immune rejection-related risks, reduces the possibilities of disease transmission, and is rich in various growth factors with natural bone-inducing activity required for bone formation. Allogeneic cancellous bone has a three-dimensional porous structure of natural bone tissue, which could be optimized by bone induction and bone conduction to promote the formation of new bone tissue in the bone graft area. Bone grafts could gradually absorb and degrade and be replaced by new bone tissue to achieve fusion. However, it is difficult to maintain an intact structure to resist axial loads, making it less ideal for maintaining intervertebral height and physiological curvature [88]. (2) Metallic materials, mainly including titanium alloys. These have excellent biomechanical properties and good early stability. At the same time, cell adhesion and osseointegration capacity could be improved by metal surface modification, making them suitable for 3D-printed personalized interbody fusion cages. Especially in recent years, the vigorous development of the 3D printing additive manufacturing industry has led to significant advancements in personalized defect repair and creation of porous structure alloys with pre-controlled porosity. At present, TC4(Ti6Al4V) biphase alloy material is the main force and the porous structure cage manufactured by 3D printing has achieved good clinical efficacy. The porous intervertebral implants have the following characteristics [89,90]: ① good biocompatibility; ② the interpenetrating micropore structure is conducive to cell adhesion and bone tissue growth and facilitates nutrient transport and metabolism; ③ While maintaining the biomechanical stiffness requirements, it further reduces the overall mass and stress shielding effect and meets the requirements of the anatomical load. However, they have a high elastic modulus, stress shielding, a risk of cage subsidence, occupy effective bone growth space, and are difficult to assess internal bone fusion status on X-ray examination. Additionally, CT and MRI examinations may exhibit metal artifacts. The generation of metal debris may cause long-term bone resorption [91,92]. (3) Polymeric materials, including polyetheretherketone (PEEK) and carbon fiber. Their characteristics include an elastic modulus close to bone tissue, mechanical properties similar to cortical bone, minimal stress shielding, good translucency, and favorable imaging observation. With the wide application of PEEK material in clinical practice, some shortcomings are also exposed: ① Because the PEEK surface is smooth and has fewer hydrophilic groups, it can not provide an ideal osteogenic environment for cell adhesion, which might lead to poor bone integration ability; ② PEEK, as a kind of bioinert material, has poor antibacterial performance. In order to optimize the above shortcomings and improve the fusion rate, some researchers have studied the surface modifications of PEEK materials by biomimetic processing, including adding carbon fiber, nano-hydroxyapatite, titanium coating, and other modification methods [93,94,95], so as to further optimize and improve the mechanical properties and osteogenic induction ability. At present, in spinal surgery, carbon fiber is mostly used in resin materials with modified materials and poor biomechanics to optimize and improve its mechanical properties. (4) Biodegradable polymeric materials, such as polycarbonate and α-polylactic acid. Most of the absorbable materials have excellent histocompatibility. They can be gradually broken down into non-toxic small molecules over time in the body, which can be absorbed, utilized by tissues, or participate in the metabolism of substances in the body [96]. Under ideal conditions, cage degradation begins slowly after 2 months of intervertebral implantation, is gradually metabolized in 12–18 months, and finally decomposes into carbon dioxide and water [97]. The degradation rate is roughly in line with the normal bone tissue reconstruction and repair time. In vivo, cage degradation and the spinal fusion process intersect and occur simultaneously. The regenerated bone tissue gradually replaces the polylactic acid material fusion device until finally, bone fusion is achieved. High concentrations of degradation products (acidic and crystalline components) can lead to severe tissue reactions, such as infection and bone resorption, limiting their clinical application [98]. The ideal material for interbody fusion cages should have the following characteristics: (1) Good biocompatibility; (2) Stable chemical properties; (3) Biomechanical properties similar to bone tissue; (4) Promotion of bone fusion; (5) Favorable for imaging observation; and (6) Biodegradability.

## 5. Conclusions

With the development of mechanisms regarding osteobiology, endochondral ossification has been an essential factor in achieving spinal fusion. Firstly, the longitudinal growth of the spine relies on secondary ossification centers, particularly the proliferation and differentiation of chondrocytes in the epiphyseal GP. Chondrocytes within the epiphysis undergo multi-stage differentiation, from resting phase to proliferative phase, prehypertrophic phase, hypertrophic phase, and finally terminal differentiation, ultimately promoting bone formation. Secondly, the detailed mechanisms of repairing bone injuries through endochondral ossification after trauma are discussed, including acute inflammatory response, recruitment of MSCs, formation of cartilaginous callus, vascular reconstruction, mineralization and absorption of cartilaginous callus, and bone remodeling. These processes are intricately regulated by various hormones, signaling pathways, and cytokines, ensuring effective repair of bone injuries. This article not only provides an in-depth biological perspective on spinal fusion but also offers new insights into the treatment of related diseases. In the future, using cartilage-derived bone-like constructions offers promise in enhancing bony fusion after surgery.

## Data Availability

No new data were created or analyzed in this study.

## Abbreviations

DS: degenerative scoliosis; LIF: lumbar interbody fusion; PLIF: posterior lumbar interbody fusion; BMP: bone morphogenetic protein; GP: growth plates; MSCs: mesenchymal cells; ECM: extracellular matrix; TGF-β: transforming growth factor-β; FGF: fibroblast growth factor; HH: Hedgehog; TNF-α: tumor necrosis factor-α; IL-1β: interleukin-1β; VEGF: vascular endothelial growth factor; SDF-1: stromal cell-derived factor 1; CXCR-4: cells expressing chemokine receptor-4; HCs: hypertrophic chondrocytes; EGFR: epidermal growth factor receptor; ROS: reactive oxygen species; MMPs: metalloproteinases; M-CSF: macrophage colony-stimulating factor; OPG: osteoprotegerin; PTH: parathyroid hormone; PTHrP: parathyroid hormone-related peptide; TLIF: transforaminal lumbar interbody fusion; PEEK: polyetheretherketone.

## References

[1] Petrosyan E., Fares J., Lesniak M.S., Koski T.R., El Tecle N.E. (2023). Biological principles of adult degenerative scoliosis. Trends Mol. Med..

[2] Chen K., Zhao J., Yang Y., Wei X., Chen Z., Li M., Zhai X. (2020). Global research trends of adult degenerative scoliosis in this decade (2010–2019): A bibliometric study. Eur. Spine J..

[3] de Kunder S.L., Rijkers K., Caelers I.J.M.H., de Bie R.A., Koehler P.J., van Santbrink H. (2018). Lumbar Interbody Fusion: A Historical Overview and a Future Perspective. Spine.

[4] Zhu H., Zhong W., Zhang P., Liu X., Huang J., Liu F., Li J. (2020). Biomechanical evaluation of autologous bone-cage in posterior lumbar interbody fusion: A finite element analysis. BMC Musculoskelet. Disord..

[5] Scott-Young M., Nielsen D., Riar S. (2023). Fundamentals of Mechanobiology and Potential Applications in Spinal Fusion. Int. J. Spine Surg..

[6] Rao P.J., Pelletier M.H., Walsh W.R., Mobbs R.J. (2014). Spine interbody implants: Material selection and modification, functionalization and bioactivation of surfaces to improve osseointegration. Orthop. Surg..

[7] Xie M., Gol’Din P., Herdina A.N., Estefa J., Medvedeva E.V., Li L., Newton P.T., Kotova S., Shavkuta B., Saxena A. (2020). Secondary ossification center induces and protects growth plate structure. Elife.

[8] Tong W., Tower R.J., Chen C., Wang L., Zhong L., Wei Y., Sun H., Cao G., Jia H., Pacifici M. (2019). Periarticular Mesenchymal Progenitors Initiate and Contribute to Secondary Ossification Center Formation During Mouse Long Bone Development. Stem Cells.

[9] Michigami T. (2013). Regulatory mechanisms for the development of growth plate cartilage. Cell. Mol. Life Sci..

[10] Burdan F., Szumiło J., Korobowicz A., Farooquee R., Patel S., Patel A., Dave A., Szumiło M., Solecki M., Klepacz R. (2009). Morphology and physiology of the epiphyseal growth plate. Folia Histochem. Cytobiol..

[11] Percival C.J., Richtsmeier J.T. (2013). Angiogenesis and intramembranous osteogenesis. Dev. Dyn..

[12] Melrose J., Shu C., Whitelock J.M., Lord M.S. (2016). The cartilage extracellular matrix as a transient developmental scaffold for growth plate maturation. Matrix Biol..

[13] Pazzaglia U.E., Reguzzoni M., Pagani F., Sibilia V., Congiu T., Salvi A.G., Benetti A. (2018). Study of Endochondral Ossification in Human Fetalcartilage Anlagen of Metacarpals: Comparative Morphology of Mineral Deposition in Cartilage and in the Periosteal Bone Matrix. Anat. Rec..

[14] Long F., Ornitz D.M. (2013). Development of the endochondral skeleton. Cold Spring Harb. Perspect. Biol..

[15] Janicki P., Kasten P., Kleinschmidt K., Luginbuehl R., Richter W. (2010). Chondrogenic pre-induction of human mesenchymal stem cells on beta-TCP: Enhanced bone quality by endochondral heterotopic bone formation. Acta Biomater..

[16] He J., Jiang B., Dai Y., Hao J., Zhou Z., Tian Z., Wu F., Gu Z. (2013). Regulation of the osteoblastic and chondrocytic differentiation of stem cells by the extracellular matrix and subsequent bone formation modes. Biomaterials.

[17] He J., Meng G., Yao R., Jiang B., Wu Y., Wu F. (2016). The essential role of inorganic substrate in the migration and osteoblastic differentiation of mesenchymal stem cells. J. Mech. Behav. Biomed. Mater..

[18] Fan L., Chen J., Tao Y., Heng B.C., Yu J., Yang Z., Ge Z. (2019). Enhancement of the chondrogenic differentiation of mesenchymal stem cells and cartilage repair by ghrelin. J. Orthop. Res..

[19] Griffin M., Hindocha S., Khan W.S. (2012). Chondrogenic differentiation of adult MSCs. Curr. Stem Cell Res. Ther..

[20] Sandell L.J., Sugai J.V., Trippel S.B. (1994). Expression of collagens I, II, X, and XI and aggrecan mRNAs by bovine growth plate chondrocytes in situ. J. Orthop. Res..

[21] Mwale F., Billinghurst C., Wu W., Alini M., Webber C., Reiner A., Ionescu M., Poole J., Poole A.R. (2000). Selective assembly and remodelling of collagens II and IX associated with expression of the chondrocyte hypertrophic phenotype. Dev. Dyn..

[22] Xiao Z.F., Su G.Y., Hou Y., Chen S.D., Lin D.K. (2018). Cartilage degradation in osteoarthritis: A process of osteochondral remodeling resembles the endochondral ossification in growth plate?. Med. Hypotheses.

[23] Fang R., Haxaire C., Otero M., Lessard S., Weskamp G., McIlwain D.R., Mak T.W., Lichtenthaler S.F., Blobel C.P. (2020). Role of iRhoms 1 and 2 in Endochondral Ossification. Int. J. Mol. Sci..

[24] Tarantino R., Chiu L.L.Y., Weber J.F., Tse M.Y., Bardana D.D., Pang S.C., Waldman S.D. (2021). Effect of nutrient metabolism on cartilaginous tissue formation. Biotechnol. Bioeng..

[25] Kobayashi T., Chung U.-I., Schipani E., Starbuck M., Karsenty G., Katagiri T., Goad D.L., Lanske B., Kronenberg H.M. (2002). PTHrP and Indian hedgehog control differentiation of growth plate chondrocytes at multiple steps. Development.

[26] Liu Z., Mohan S., Yakar S. (2016). Does the GH/IGF-1 axis contribute to skeletal sexual dimorphism? Evidence from mouse studies. Growth Horm. IGF Res..

[27] Li J., Dong S. (2016). The Signaling Pathways Involved in Chondrocyte Differentiation and Hypertrophic Differentiation. Stem Cells Int..

[28] Green J.D., Tollemar V., Dougherty M., Yan Z., Yin L., Ye J., Collier Z., Mohammed M.K., Haydon R.C., Luu H.H. (2015). Multifaceted signaling regulators of chondrogenesis: Implications in cartilage regeneration and tissue engineering. Genes. Dis..

[29] Cleary M.A., van Osch G.J.V.M., Brama P.A., Hellingman C.A., Narcisi R. (2015). FGF, TGFβ and Wnt crosstalk: Embryonic to in vitro cartilage development from mesenchymal stem cells. J. Tissue Eng. Regen. Med..

[30] Thielen N.G.M., van der Kraan P.M., van Caam A.P.M. (2019). TGFβ/BMP Signaling Pathway in Cartilage Homeostasis. Cells.

[31] Maes C. (2017). Signaling pathways effecting crosstalk between cartilage and adjacent tissues: Seminars in cell and developmental biology: The biology and pathology of cartilage. Semin. Cell Dev. Biol..

[32] Racine H.L., Serrat M.A. (2020). The Actions of IGF-1 in the Growth Plate and Its Role in Postnatal Bone Elongation. Curr. Osteoporos. Rep..

[33] Gentili C., Cancedda R. (2009). Cartilage and bone extracellular matrix. Curr. Pharm. Des..

[34] Prein C., Beier F. (2019). ECM signaling in cartilage development and endochondral ossification. Curr. Top. Dev. Biol..

[35] Samsa W.E., Zhou X., Zhou G. (2017). Signaling pathways regulating cartilage growth plate formation and activity. Semin. Cell Dev. Biol..

[36] Thompson E.M., Matsiko A., Kelly D.J., Gleeson J.P., O’Brien F.J. (2016). An Endochondral Ossification-Based Approach to Bone Repair: Chondrogenically Primed Mesenchymal Stem Cell-Laden Scaffolds Support Greater Repair of Critical-Sized Cranial Defects Than Osteogenically Stimulated Constructs In Vivo. Tissue Eng. Part. A.

[37] Liu Y., Kuang B., Rothrauff B.B., Tuan R.S., Lin H. (2019). Robust bone regeneration through endochondral ossification of human mesenchymal stem cells within their own extracellular matrix. Biomaterials.

[38] Fahy N., Palomares Cabeza V., Lolli A., Witte-Bouma J., Merino A., Ridwan Y., Wolvius E.B., Hoogduijn M.J., Farrell E., Brama P.A.J. (2021). Chondrogenically Primed Human Mesenchymal Stem Cells Persist and Undergo Early Stages of Endochondral Ossification in an Immunocompetent Xenogeneic Model. Front. Immunol..

[39] Herrmann M., Verrier S., Alini M. (2015). Strategies to Stimulate Mobilization and Homing of Endogenous Stem and Progenitor Cells for Bone Tissue Repair. Front. Bioeng. Biotechnol..

[40] Zigdon-Giladi H., Rudich U., Geller G.M., Evron A. (2015). Recent advances in bone regeneration using adult stem cells. World J. Stem Cells.

[41] Dennis S.C., Berkland C.J., Bonewald L.F., Detamore M.S. (2015). Endochondral ossification for enhancing bone regeneration: Converging native extracellular matrix biomaterials and developmental engineering in vivo. Tissue Eng. Part. B Rev..

[42] Yang Y., Lee E.H., Yang Z. (2022). Hypoxia-Conditioned Mesenchymal Stem Cells in Tissue Regeneration Application. Tissue Eng. Part. B Rev..

[43] Kruijt Spanjer E.C., Bittermann G.K.P., van Hooijdonk I.E.M., Rosenberg A.J.W.P., Gawlitta D. (2017). Taking the endochondral route to craniomaxillofacial bone regeneration: A logical approach?. J. Craniomaxillofac. Surg..

[44] Luyendyk J.P., Schoenecker J.G., Flick M.J. (2019). The multifaceted role of fibrinogen in tissue injury and inflammation. Blood.

[45] Kushioka J., Chow S.K.-H., Toya M., Tsubosaka M., Shen H., Gao Q., Li X., Zhang N., Goodman S.B. (2023). Bone regeneration in inflammation with aging and cell-based immunomodulatory therapy. Inflamm. Regen..

[46] Schmidt-Bleek K., Schell H., Schulz N., Hoff P., Perka C., Buttgereit F., Volk H.-D., Lienau J., Duda G.N. (2012). Inflammatory phase of bone healing initiates the regenerative healing cascade. Cell Tissue Res..

[47] Wu C.-L., Harasymowicz N., Klimak M., Collins K., Guilak F. (2020). The role of macrophages in osteoarthritis and cartilage repair. Osteoarthr. Cartil..

[48] Fan S., Sun X., Su C., Xue Y., Song X., Deng R. (2023). Macrophages-bone marrow mesenchymal stem cells crosstalk in bone healing. Front. Cell Dev. Biol..

[49] Pajarinen J., Lin T., Gibon E., Kohno Y., Maruyama M., Nathan K., Lu L., Yao Z., Goodman S.B. (2019). Mesenchymal stem cell-macrophage crosstalk and bone healing. Biomaterials.

[50] Stefanowski J., Lang A., Rauch A., Aulich L., Köhler M., Fiedler A.F., Buttgereit F., Schmidt-Bleek K., Duda G.N., Gaber T. (2019). Spatial Distribution of Macrophages During Callus Formation and Maturation Reveals Close Crosstalk between Macrophages and Newly Forming Vessels. Front. Immunol..

[51] Schmidt-Bleek K., Schell H., Lienau J., Schulz N., Hoff P., Pfaff M., Schmidt G., Martin C., Perka C., Buttgereit F. (2014). Initial immune reaction and angiogenesis in bone healing. J. Tissue Eng. Regen. Med..

[52] Liu J., Chen B., Yan F., Yang W. (2017). The Influence of Inflammatory Cytokines on the Proliferation and Osteoblastic Differentiation of MSCs. Curr. Stem Cell Res. Ther..

[53] Zhou Q., Yang C., Yang P. (2017). The Promotional Effect of Mesenchymal Stem Cell Homing on Bone Tissue Regeneration. Curr. Stem Cell Res. Ther..

[54] Shinohara K., Greenfield S., Pan H., Vasanji A., Kumagai K., Midura R.J., Kiedrowski M., Penn M.S., Muschler G.F. (2011). Stromal cell-derived factor-1 and monocyte chemotactic protein-3 improve recruitment of osteogenic cells into sites of musculoskeletal repair. J. Orthop. Res..

[55] Xu W., Xu R., Li Z., Wang Y., Hu R. (2019). Hypoxia changes chemotaxis behaviour of mesenchymal stem cells via HIF-1α signalling. J. Cell. Mol. Med..

[56] Yellowley C. (2013). CXCL12/CXCR4 signaling and other recruitment and homing pathways in fracture repair. Bonekey Rep..

[57] Yu X., Wan Q., Cheng G., Cheng X., Zhang J., Pathak J.L., Li Z. (2018). CoCl(2), a mimic of hypoxia, enhances bone marrow mesenchymal stem cells migration and osteogenic differentiation via STAT3 signaling pathway. Cell Biol. Int..

[58] Wong S.A., Rivera K.O., Miclau T., Alsberg E., Marcucio R.S., Bahney C.S. (2018). Microenvironmental Regulation of Chondrocyte Plasticity in Endochondral Repair—A New Frontier for Developmental Engineering. Front. Bioeng. Biotechnol..

[59] Aghajanian P., Mohan S. (2018). The art of building bone: Emerging role of chondrocyte-to-osteoblast transdifferentiation in endochondral ossification. Bone Res..

[60] Gandica Y., Schwarz T., Oliveira O., Travasso R.D.M. (2014). Hypoxia in vascular networks: A complex system approach to unravel the diabetic paradox. PLoS ONE.

[61] Yang Y., Lin H., Shen H., Wang B., Lei G., Tuan R.S. (2018). Mesenchymal stem cell-derived extracellular matrix enhances chondrogenic phenotype of and cartilage formation by encapsulated chondrocytes in vitro and in vivo. Acta Biomater..

[62] Mao Y., Block T., Singh-Varma A., Sheldrake A., Leeth R., Griffey S., Kohn J. (2019). Extracellular matrix derived from chondrocytes promotes rapid expansion of human primary chondrocytes in vitro with reduced dedifferentiation. Acta Biomater..

[63] Bardsley K., Kwarciak A., Freeman C., Brook I., Hatton P., Crawford A. (2017). Repair of bone defects in vivo using tissue engineered hypertrophic cartilage grafts produced from nasal chondrocytes. Biomaterials.

[64] Bernhard J., Ferguson J., Rieder B., Heimel P., Nau T., Tangl S., Redl H., Vunjak-Novakovic G. (2017). Tissue-engineered hypertrophic chondrocyte grafts enhanced long bone repair. Biomaterials.

[65] Hu K., Olsen B.R. (2016). The roles of vascular endothelial growth factor in bone repair and regeneration. Bone.

[66] Hu K., Olsen B.R. (2017). Vascular endothelial growth factor control mechanisms in skeletal growth and repair. Dev. Dyn..

[67] Hu K., Olsen B.R. (2016). Osteoblast-derived VEGF regulates osteoblast differentiation and bone formation during bone repair. J. Clin. Investig..

[68] Kim P., Park J., Lee D.-J., Mizuno S., Shinohara M., Hong C.P., Jeong Y., Yun R., Park H., Park S. (2022). Mast4 determines the cell fate of MSCs for bone and cartilage development. Nat. Commun..

[69] Zhou X., von der Mark K., Henry S., Norton W., Adams H., de Crombrugghe B. (2014). Chondrocytes transdifferentiate into osteoblasts in endochondral bone during development, postnatal growth and fracture healing in mice. PLoS Genet..

[70] Qin X., Jiang Q., Nagano K., Moriishi T., Miyazaki T., Komori H., Ito K., von der Mark K., Sakane C., Kaneko H. (2020). Runx2 is essential for the transdifferentiation of chondrocytes into osteoblasts. PLoS Genet..

[71] Qin X., Jiang Q., Komori H., Sakane C., Fukuyama R., Matsuo Y., Ito K., Miyazaki T., Komori T. (2021). Runt-related transcription factor-2 (Runx2) is required for bone matrix protein gene expression in committed osteoblasts in mice. J. Bone Miner. Res..

[72] Wu C.W., Tchetina E.V., Mwale F., Hasty K., Pidoux I., Reiner A., Chen J., van Wart H.E., Poole A.R., Poole D.A.R. (2002). Proteolysis involving matrix metalloproteinase 13 (collagenase-3) is required for chondrocyte differentiation that is associated with matrix mineralization. J. Bone Miner. Res..

[73] Schlundt C., El Khassawna T., Serra A., Dienelt A., Wendler S., Schell H., van Rooijen N., Radbruch A., Lucius R., Hartmann S. (2018). Macrophages in bone fracture healing: Their essential role in endochondral ossification. Bone.

[74] Zhang W., Ling C., Li X., Sheng R., Liu H., Zhang A., Jiang Y., Chen J., Yao Q. (2020). Cell-Free Biomimetic Scaffold with Cartilage Extracellular Matrix-Like Architectures for In Situ Inductive Regeneration of Osteochondral Defects. ACS Biomater. Sci. Eng..

[75] Gamblin A.L., Renaud A., Charrier C., Hulin P., Louarn G., Heymann D., Trichet V., Layrolle P. (2014). Osteoblastic and osteoclastic differentiation of human mesenchymal stem cells and monocytes in a miniaturized three-dimensional culture with mineral granules. Acta Biomater..

[76] Kameo Y., Adachi T., Hojo M. (2011). Effects of loading frequency on the functional adaptation of trabeculae predicted by bone remodeling simulation. J. Mech. Behav. Biomed. Mater..

[77] Riquelme M.A., Cardenas E.R., Xu H., Jiang J.X. (2020). The Role of Connexin Channels in the Response of Mechanical Loading and Unloading of Bone. Int. J. Mol. Sci..

[78] Kenkre J.S., Bassett J.H.D. (2018). The bone remodelling cycle. Ann. Clin. Biochem..

[79] Katsimbri P. (2017). The biology of normal bone remodelling. Eur. J. Cancer Care.

[80] Guihard P., Danger Y., Brounais B., David E., Brion R., Delecrin J., Richards C.D., Chevalier S., Rédini F., Heymann D. (2012). Induction of osteogenesis in mesenchymal stem cells by activated monocytes/macrophages depends on oncostatin M signaling. Stem Cells.

[81] Udagawa N., Koide M., Nakamura M., Nakamichi Y., Yamashita T., Uehara S., Kobayashi Y., Furuya Y., Yasuda H., Fukuda C. (2021). Osteoclast differentiation by RANKL and OPG signaling pathways. J. Bone Miner. Metab..

[82] Suda T., Udagawa N., Nakamura I., Miyaura C., Takahashi N. (1995). Modulation of osteoclast differentiation by local factors. Bone.

[83] Hinton R., Jing Y., Jing J., Feng J. (2017). Roles of Chondrocytes in Endochondral Bone Formation and Fracture Repair. J. Dent. Res..

[84] Stegen S., Laperre K., Eelen G., Rinaldi G., Fraisl P., Torrekens S., van Looveren R., Loopmans S., Bultynck G., Vinckier S. (2019). HIF-1α metabolically controls collagen synthesis and modification in chondrocytes. Nature.

[85] Sielatycki J.A., Saito M., Yuasa M., Moore-Lotridge S.N., Uppuganti S., Colazo J.M., Hysong A.A., Robinette J.P., Okawa A., Yoshii T. (2018). Autologous chondrocyte grafting promotes bone formation in the posterolateral spine. JOR Spine.

[86] Chung C.G., James A.W., Asatrian G., Chang L., Nguyen A., Le K., Bayani G., Lee R., Stoker D., Pang S. (2014). Human perivascular stem cell-based bone graft substitute induces rat spinal fusion. Stem Cells Transl. Med..

[87] de Kunder S.L., van Kuijk S.M.J., Rijkers K., Caelers I.J.M.H., van Hemert W.L.W., de Bie R.A., van Santbrink H. (2017). Transforaminal lumbar interbody fusion (TLIF) versus posterior lumbar interbody fusion (PLIF) in lumbar spondylolisthesis: A systematic review and meta-analysis. Spine J..

[88] Haugen H.J., Lyngstadaas S.P., Rossi F., Perale G. (2019). Bone grafts: Which is the ideal biomaterial?. J. Clin. Periodontol..

[89] McGilvray K.C., Easley J., Seim H.B., Regan D., Berven S.H., Hsu W.K., Mroz T.E., Puttlitz C.M. (2018). Bony ingrowth potential of 3D-printed porous titanium alloy: A direct comparison of interbody cage materials in an in vivo ovine lumbar fusion model. Spine J..

[90] Vlad M.D., Aguado E.F., González S.G., Ivanov I.C., Şindilar E.V., Poeată I., Iencean A.Ş., Butnaru M., Avădănei E.R., López J.L. (2020). Novel titaniumapatite hybrid scaffolds with spongy bone-like micro architecture intended for spinal application: In vitro and in vivo study. Mater. Sci. Eng. C Mater. Biol. Appl..

[91] Fan L., Chen S., Yang M., Liu Y., Liu J. (2024). Metallic Materials for Bone Repair. Adv. Healthcare Mater..

[92] Liang W., Zhou C., Zhang H., Bai J., Jiang B., Jiang C., Ming W., Zhang H., Long H., Huang X. (2023). Recent advances in 3D printing of biodegradable metals for orthopaedic applications. J. Biol. Eng..

[93] Kashii M., Kitaguchi K., Makino T., Kaito T. (2020). Comparison in the same intervertebral space between titanium-coated and uncoated PEEK cages in lumbar interbody fusion surgery. J. Orthop. Sci..

[94] Wang S., Deng Y., Yang L., Shi X., Yang W., Chen Z.-G. (2018). Enhanced antibacterial property and osteodifferentiation activity on plasma treated porous polyetheretherketone with hierarchical micro/nano-topography. J. Biomater. Sci. Polym. Ed..

[95] Xue Z., Wang Z., Sun A., Huang J., Wu W., Chen M., Hao X., Huang Z., Lin X., Weng S. (2020). Rapid construction of polyetheretherketone (PEEK) biological implants incorporated with brushite (CaHPO(4)·2H(2)O) and antibiotics for anti-infection and enhanced osseointegration. Mater. Sci. Eng. C.

[96] Hojo Y., Kotani Y., Ito M., Abumi K., Kadosawa T., Shikinami Y., Minami A. (2005). A biomechanical and histological evaluation of a bioresorbable lumbar interbody fusion cage. Biomaterials.

[97] Lowe T.G., Coe J.D. (2002). Resorbable polymer implants in unilateral transforaminal lumbar interbody fusion. J. Neurosurg. Spine.

[98] Wang J., Wang L., Zhou Z., Lai H., Xu P., Liao L., Wei J. (2016). Biodegradable Polymer Membranes Applied in Guided Bone/Tissue Regeneration: A Review. Polymers.

